# Dietary iron interacts with diet composition to modulate the endocannabinoidome and the gut microbiome in mice

**DOI:** 10.1017/gmb.2025.1

**Published:** 2025-02-14

**Authors:** Fredy Alexander Guevara Agudelo, Nadine Leblanc, Isabelle Bourdeau-Julien, Gabrielle St-Arnaud, Fadil Dahhani, Nicolas Flamand, Alain Veilleux, Vincenzo Di Marzo, Frédéric Raymond

**Affiliations:** 1 Centre Nutrition, santé et société (NUTRISS), et Institut sur la Nutrition et les aliments fonctionnels (INAF), Université Laval, Québec, Canada; 2 Chaire d’excellence en recherche du Canada sur l’axe microbiome – endocannabinoidome dans la santé métabolique (CERC-MEND), Québec, Canada; 3 École de nutrition, Faculté des sciences de l’agriculture et de l’alimentation, Université Laval, Québec, Canada; 4 Faculté de médecine, Institut universitaire de cardiologie et pneumologie de Québec, Université Laval, Québec, Canada

**Keywords:** endocannabinoid (eCB) system, inflammation, intestine, iron, metabolic health, microbiome, nutrition

## Abstract

The endocannabinoidome (eCBome) and the gut microbiota have been implicated in diet-induced obesity and impaired metabolism. While the eCBome and the gut microbiome are known to respond to diet macronutrient composition, interaction with micronutrient intake has been relatively unexplored. Iron (Fe) is an essential micronutrient for the function of enzymes involved in energy and lipid metabolism. Here, we evaluated how 28 days of Fe depletion and enrichment, in interaction with Low Fat-Low Sucrose (LFLS) or High Fat-High Sucrose (HFHS) diets, affect the host via the eCBome, and modulate intestinal gut microbial communities. Circulating levels of *N*-oleoyl-ethanolamine (OEA) showed an elevation associated with Fe-enriched LFLS diet, while the Fe-depleted HFHS diet showed an elevation of *N*-arachidonoyl-ethanolamine (anandamide, AEA) and a decrease of circulating linoleic acid. In parallel, the response of intestinal inflammatory mediators to Fe in the diet showed decreased levels of prostaglandins PGE_1_, PGE_3_, and 1a,1b-dihomo PGF_2_α in the caecum. Individual differences in microbial taxa were less pronounced in the ileum than in the caecum, where *Eubacterium coprostanoligenes* group showed an increase in relative abundance associated with Fe-depleted LFLS diets. In conclusion, our study shows that Fe intake modulates the response to the macronutrient composition of the diet in mice.

## Introduction

Iron (Fe) is a fundamental micronutrient that plays a role in oxygen transport (Lakhal-Littleton and Robbins, 2017), the synthesis of metabolic enzymes (Cerami, 2017), cellular respiration (Oexle et al., 1999), and the maintenance of normal immune function (Ni et al., 2022). Fe mediates electron transfer and oxygen supply in oxidation–reduction reactions which, although vital for maintaining normal cellular metabolism, can also result in the generation of toxic reactive oxygen species ROS (Hentze et al., 2004). Fe is obtained from the diet in different forms and can be classified into two types: heme-Fe and non-heme Fe. Heme-Fe is found mainly in animal products and is the most bioavailable form, with absorption rates between 15% and 35%. This form of Fe is readily absorbed but accounts for only 5%–10% in most diets.

Obesity is associated with low-grade chronic inflammation (Ellulu et al., 2017) and over the last few years, it has been reported that this condition alters Fe metabolism. In adults and children, obesity is linked to hypoferraemia, impaired Fe absorption, and lower Fe stores despite adequate dietary Fe intake (Baumgartner et al., 2013). In particular, individuals with obesity or combined chronic inflammatory diseases are more likely to have hypoferraemia, which could be associated with Fe deprivation caused by the inflammatory response (Yanoff et al., 2007). Reduced plasma ferritin has been previously observed to improve nonalcoholic fatty liver disease in individuals with obesity, suggesting that it is essential to consider the Fe status in the treatment of obesity-related metabolic dysfunction (Moore Heslin et al., 2021). Recent studies have emphasized the importance of Fe in the regulation of lipid homeostasis (Rodríguez-Pérez et al., 2018). Indeed, both Fe insufficiency, especially in severe obesity (Aigner et al., 2014) and Fe overload syndrome has been well-studied in association with obesity-related diseases (Moore Heslin et al., 2021).

Host-microbiota interactions are directly influenced by Fe, which alters bacterial growth in the intestine. Both deficiency and excess of Fe are important in terms of gut microbiota dysbiosis. Dysbiosis has been associated with a number of human diseases, such as autoimmune disorders (Collado et al., 2015), increased vulnerability to cancers (Viaud et al., 2014), irritable bowel syndrome (Kostic et al., 2014), and the progression of obesity (Boulangé et al., 2016). Gut microbiota and their metabolites could potentially exert an influence on inflammatory conditions in the host (Feng et al., 2018). Indeed, it is well known that gut microbiota plays a major role in the development of food absorption and low-grade inflammation (Al Bander et al., 2020). Although there are increased amounts of dietary Fe in the colon, bacteria may still compete to incorporate Fe due to the formation of Fe complexes with other food components and the low solubility of ferric Fe due to a higher pH in the colon (Kortman et al., 2014). Immune-mediated inflammatory diseases, such as Crohn’s disease (CD), ulcerative colitis (UC), multiple sclerosis (MS), and rheumatoid arthritis (RA), modify the composition of the gut microbiota and Fe has also been linked to the development of these diseases (Kaitha et al., 2015).

Fe is an essential cofactor for peroxidase, lipoxygenase, and cyclooxygenase enzymes involved in the catabolism of arachidonic acid. Arachidonic acid (AA) plays essential roles, especially in cell signalling through its role as a precursor of numerous eicosanoids such as prostaglandins and leukotrienes. Indeed, previous studies have shown that Fe-citrate but not sodium citrate (Na-citrate) downregulates the production of PGE_2_ (Hisakawa et al., 1998). Moreover, AA is also a molecular block of the endocannabinoids 2-arachidonoyl-glycerol (2-AG) and *N*-arachidonoyl-ethanolamine (Anandamide or AEA), that have signalling functions in appetite regulation and energy metabolism, in relation to the modulation of neurotransmitter release (Almeida et al., 2022), which could involve physiological and pathophysiological phenomena.

The endocannabinoid system (eCBs) is a signalling system comprised of endogenous lipids mediators, the endocannabinoids AEA (also known as anandamide) and 2-AG, which bind to two G protein-coupled receptors, the cannabinoid type 1 and type 2 (CB_1_ and CB_2_) receptors, expressed throughout the body. The endocannabinoidome (eCBome) is defined as an extension of the eCBs that also includes the congeners of AEA and 2-AG, the *N*-acyl-ethanolamines (NAEs) and 2-monoacyl-glycerols (2-MAGs), respectively, together with additional enzymes and receptors related to these molecules (Iannotti and Di Marzo, 2021). Endocannabinoids and their congeners are synthesized from membrane phospholipid precursors containing the corresponding fatty acids either esterified to the 2-hydroxy group of glycerol in, usually, phosphatidylinositol, for 2-MAGs, or amidated by the NH_2_-group of phosphatidylethanolamine, for NAEs (Simard et al., 2022). The eCBome is involved in several physiological processes such as satiety, energy control, and other essential functions in metabolic health (Silvestri and Di Marzo, 2013). For instance, *N*-oleoyl-ethanolamine (OEA) can inhibit food intake, while palmitoylethanolamide (PEA) has anti-inflammatory activity through the activation of several receptors including peroxisome proliferator-activated receptor α (PPARα) (Alhouayek and Muccioli, 2014). The eCBome mediates a number of physiological and pathophysiological responses in the intestine via activation of the cannabinoid receptors, TRPV channels, and several GPR, for example, maintaining homeostasis in the gut by controlling hypercontractility and permeability, and promoting regeneration after injury (Taschler et al., 2017). The small intestine serves both as an organ for digestion and absorption of food, and for signalling to the brain and peripheral organs about the amount of incoming food (Psichas et al., 2015). NAEs and 2-MAGs may participate in the regulation of gut–brain signalling in relation to the control of food intake.

The crosstalk between the intestinal eCBome and gut microbiota regulates many gastrointestinal functions, such as hormone secretion, intestinal permeability, motility, immune response, and nutrient absorption (Cuddihey et al., 2022). There are numerous environmental and host genetic factors that can impact on the structure of the intestinal microbiota, but diet is considered to be the main driver (Moles and Otaegui, 2020). Studies have been focused on exploring the impact of macronutrients, such as carbohydrates and proteins, on colonic and faecal bacterial populations (Castonguay-paradis et al., 2020; Rowland et al., 2018). However, there have been substantially fewer investigations on the modulatory effects of micronutrients. Previous studies have demonstrated that lipid mediators, including the eCBome, can be modulated by micronutrients in interaction with the macronutrient composition of the diet (Guevara Agudelo et al., 2022).

In this work, we investigated how diets depleted (12 mg/kg) or enriched (150 mg/kg) in Fe modulate the eCBome and gut microbiome response to a Low Fat-Low Sucrose (LFLS) or High Fat-High Sucrose (HFHS) diet in an obesity mice model. Intake of Fe was chosen to represent low and high Fe consumption without inducing deficiency or toxicity, thus covering the full range of safe dietary intake (Asperti et al., 2018; B R Blakley, 1988; Nutrition, N. R. C. (US) Subcommittee on Laboratory Animal Nutrition, 1995). Our hypothesis is that the host response to diet macronutrient composition will be affected by Fe intake. Specifically, we studied the response of the circulating eCBome, as well as the ileum and caecum eCBome, microbiome, and inflammation mediators. Special attention is given to the intestinal response to dietary conditions, as the intestine is the first organ to be exposed to the diet. Experiments were conducted with male and female mice to examine the impact of Fe and diet formulation on gut microbiota and eCBome. The data were then stratified to assess the influence of sex on the responses of mice to Fe. Our results highlight the complexity of studying dietary components, as many interactions were observed between Fe intake and diet macronutrient composition.

## Materials and methods

### Animals, diets, and housing

The study was approved by the Université Laval animal ethics committee (CPAUL 2019-006). Forty-eight 6-week-old C57BL/6 J male and female mice were purchased from Jackson Laboratory (USA) and were individually housed in the animal facility of the Institute of Nutrition and Functional Foods (INAF), in standard cages under controlled temperature (22 °C) and relative humidity (50%) with a 12 h day/night cycle. At arrival, all mice were acclimated to their new environment for a one-week adaptation period, during which they received a normal chow diet (AIN-93G-purified diet #110700, Dyets Inc., Bethlehem, PA, USA). Following this time, mice were randomly assigned to four groups (n = 12 per group, 6 males and 6 females). The groups were defined according to four diet designs. Supplementary Table S1 presents the formulation for the four diet groups set as follows: Enriched (150 mg/kg) and depleted (12 mg/kg) concentrations of Fe in combination with High Fat-High Sucrose (HFHS: 23.6% fat, 17% sucrose, Research Diets Inc., NJ, USA), and Low Fat-Low Sucrose (LFLS: 4.3% fat, 7% sucrose, Research Diets Inc., NJ, USA). In this study, Fe was provided as ferric citrate. The diets were formulated to be isonitrogenous, although different in energy and lipid content between HFHS and LFLS. Total energy in diets was determined with an adiabatic Parr 6300 calorimeter (Parr Instrument Company, Moline, IL, USA) and was similar among LFLS and among HFHS diets (Fe-depleted LFLS 3967.15 cal/g; Fe-enriched LFLS 3906.3 cal/g; Fe-depleted HFHS 4936.85 cal/g; Fe-enriched HFHS 4886.9 cal/g). Dietary protein content was determined by combustion (Dumas method) using a LECO FP-528 apparatus (LECO Corporation, St. Joseph, MI, USA) and was 14.75% [w/w] for Fe-depleted LFLS, 14.31% for Fe-enriched LFLS, 18.79% for Fe-depleted HFHS, and 18.16% for Fe-enriched HFHS. Dietary fat content was measured with an ANKOMXT10 Extractor (ANKOM Technology, Macedon, NY, USA) and was different between the diets, reflecting the fact that we have low-fat and high-fat diet (Fe-depleted 12 mg/kg LFLS 3.81% [w/w]; Fe-enriched 150 mg/kg LFLS 4.25% [w/w]; Fe-depleted 12 mg/kg HFHS 21.62% [w/w]; Fe-enriched 150 mg/kg HFHS 22.13% [w/w]). Animals were fed *ad libitum* with these diets for 28 days and had access to *ad libitum* water. Body weight and food intake were monitored twice weekly. Mice were killed by cardiac puncture. Whole blood was collected in K3-EDTA tubes to obtain plasma (1780*g*, 10 min). Ileum and caecum tissues were collected at 10 and 2 cm from the ileocaecal junction, respectively. Luminal contents were collected in PBS by gentle scraping. Tissue samples from both ileum and caecum were treated with RNAlater Stabilization Solution (ThermoFisher, USA) to preserve the integrity of RNA until its subsequent extraction. All samples were stored at −80 °C until further analysis.

### Endocannabinoidome quantification

Lipids were extracted from plasma samples (40 μL) as in (Turcotte et al., 2020). In brief, plasma samples were diluted to a volume 500 μL with Tris buffer (50 mM, pH = 7). A total of 5 μL of deuterated standards were added to each sample and then vortexed. Two millilitres of toluene were then added, and samples were vortexed for 30 s. Samples were next placed in a dry ice-ethanol bath to freeze the aqueous phase. The toluene phase was then collected and evaporated to dryness under a stream of nitrogen. Ileum and caecum samples (5–10 mg) were extracted and processed exactly as in (Manca et al., 2020). All lipid extracts were then resuspended with 60 μL of mobile phases (50% Solvent A and 50% solvent B) and then injected (40 μL) on the injected onto an HPLC column (Kinetex C8, 150 × 2.1 mm, 2.6 μM; Phenomenex) as described before (Everard et al., 2019). Quantification of eCBome-related mediators was performed using a Shimadzu 8050 triple quadrupole mass spectrometer. The following metabolites were quantified: 1/2-oleoyl-glycerol (2-OG), 1/2-linoleoyl-glycerol (LG), 1/2-arachidonoylglycerol (2-AG), 1/2-eicosapentaenoyl-glycerol (2-EPG), 1/2-docosapentaenoyl(n-3)-glycerol (2-DPG), 2-docosahexaenoylglycerol (1/2-DHG), *N*-palmitoyl-ethanolamine (PEA), *N*-stearoylethanolamine (SEA), *N*-oleoyl-ethanolamine (OEA), *N*-linoleoyl-ethanolamine (LEA), *N*-arachidonoyl-ethanolamine (AEA), *N*-eicosapentaenoyl-ethanolamine (EPEA), *N*-docosapentaenoyl-ethanolamine (DPEA), *N*-docosahexaenoyl-ethanolamine (DHEA), arachidonic acid (AA), docosahexaenoic acid (DHA), docosaepentaenoic acid (DPA), eicosapentaenoic acid (EPA), stearidonic acid (SDA), linoleic acid (LA), PGD_2_, PGE_1_, PGE_2_, PGE_3_, 1a,1b-dihomo PGF_2α_ (1a,1b-dihomo PGF_2α_), thromboxane B_2_ (TBX), *N*-Palmitoyl-Glycine and *N*-Oleoyl-Serotonin. For the MAGs, the signals from the *sn*-1(3) and the *sn*-2 isomers were combined and presented as 2-MAGs, to take into account the rapid isomerization of the *sn*-2 isomer to *sn*-1(3).

### 16S rRNA gene sequencing

Intestinal luminal contents were lysed using bead beating (0.1 mm silica beads) before enzymatic digestion with 50 mg of lysozyme and 200 U/μL mutanolysin (37 °C, 45 min). Microbial DNA was extracted using the QIAamp DNA Stool minikit (Qiagen, CA, USA), and amplification of the V3-V4 region was performed using the primers Bact-0341-b-S-17 (5′-CCTACGGGNGGCWGCAG-3′) and Bact-0785-a-A-21 (5′-GACTACHVGGGTATCTAATCC-3′) (Illumina, CA, USA). Libraries were purified using magnetic beads AMPURE XP (Beckman Coulter Canada Lp), and libraries were assessed on gel using QIAexcel (Qiagen, CA, USA). High-throughput sequencing (2- by 300-bp paired end) was performed on a MiSeq platform (Illumina, CA, USA). Sequences were processed using the DADA2 package (version 1.16.0) (Callahan et al., 2016) and associations with bacterial taxa were obtained using the Ribosomal Database Project reference database Silva version 132. Microbiome abundances were normalized using rarefaction (Rarefaction; Vegan R package). Reads were rarefied to 5000 reads to account for depth bias (McMurdie and Holmes, 2014). Samples with read count lower than 5000 but higher than 2000 reads were kept in the analysis as is. Before rarefaction, we observed 5113 ASV and after rarefaction we observed 4923 ASV. Raw sequences were deposited to SRA under accession PRJNA977215 (https://www.ncbi.nlm.nih.gov/bioproject/PRJNA977215/).

### mRNA isolation, reverse transcription, and qPCR

RNA was extracted from the ileum and caecum samples with the RNeasy Plus mini kit (Qiagen, CA, USA) according to the manufacturer’s instructions and eluted in 30 μL of UltraPure distilled water (Invitrogen, USA). RNA concentration and purity were determined by measuring the absorbance of RNA in a nanodrop at 260 and 280 nm. A total of 500 nanograms of RNA was reverse transcribed with a high-capacity cDNA reverse transcription kit (Applied Biosystems, CA, USA). We used 7500 Real-Time PCR System (Applied biosystems, CA, USA) to perform quantitative PCR to assess the expression of 2 genes associated with anti-inflammatory activity (*Il10* and *Tgfb1*) and 2 genes associated with pro-inflammatory activity (*Il1b* and *Tnfa*) with one housekeeping gene (*Hprt*). Primers and probes for TaqMan qPCR assays were purchased as commercial kits (ThermoFisher Scientific, Burlington, ON, Canada) and TaqMan assay IDs were as follows: *Hprt* (Mm03024075_m1), *Il10* (Mm01288386_m1), *Tgfb1* (Mm01178820_m1), *Il1b* (Mm00434228_m1), and *Tnfa* (Mm00443258_m1). All expression data were normalized by the threshold cycle (2^-ΔΔCT^) method using *Hprt* as an internal control (Livak and Schmittgen, 2001).

### Statistical analyses

Data are expressed as mean ± SEM. Generalized linear regression models were used to identify the effects of Fe, diet, and sex on ranked values of eCBome mediators and gut microbiome relative abundances. We used a three-way ANOVA based on a linear model that included interactions between diet formulation (LFLS vs. HFHS), Fe concentration (depleted vs. enriched), and sex of the animal (female or male). The differences were considered statistically significant with P values of P < 0.05 using contrast tests between Fe-depleted and Fe-enriched levels, LFLS and HFHS formulations, the sex of animals (female and male), and the combination between Fe levels and diet formulations. Spearman correlations were used to investigate associations between microbiome families and eCBome mediators. Adjustments for multiple testing were obtained using false-discovery rate (FDR). Analyses were performed with R software version 4.0.2. Principal-component analysis was performed using the FactoMineR R package (Lê et al., 2008). PERMANOVA was performed between two of the segments of the intestine (ileum and caecum) with 999 permutations in conjunction with Canberra distances between samples using package vegan in R (v2.5.7).

## Results

### Dietary Fe intake has no impact on weight gain

**Figure 1.**
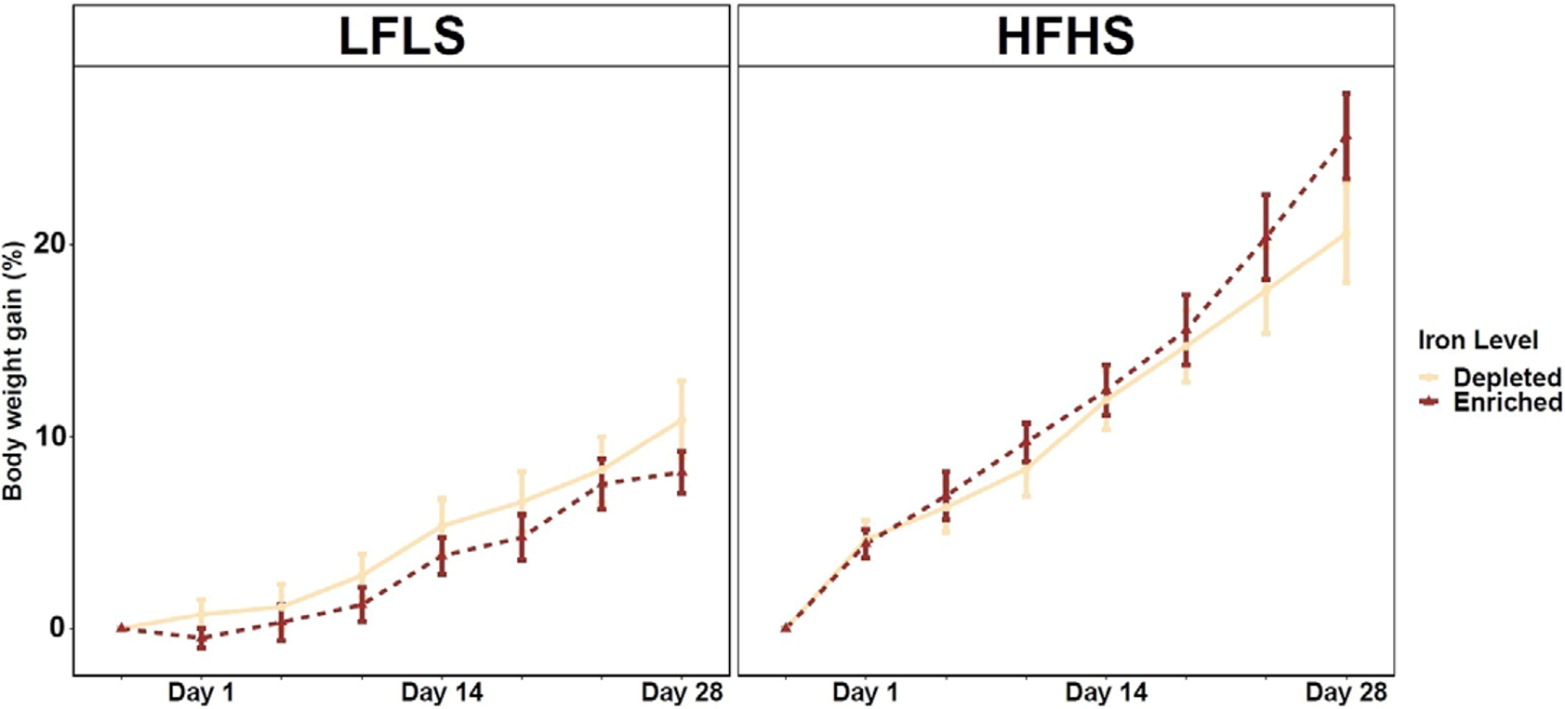
Weight gain in mice fed Fe-enriched and Fe-depleted LFLS or HFHS diets. Groups of 12 mice (6F/6M) were fed Fe-enriched /or Fe-depleted diets combined with LFLS or HFHS diet for 28 days. Generalized linear regression models were used to identify the effects of Fe and macronutrient diet formulation interactions on weight gain (%) over time for 28 days of study. Data are expressed as mean ± SEM (n = 12).

Variations in dietary Fe intake showed no clear effect on the weight gain of the mice after 28 days. However, as expected, mice fed with HFHS diets showed an increase in weight in comparison with LFLS, regardless of Fe intake (Figure 1). As for sex differences, male mice showed a greater weight gain than females on both types of Fe-enriched and Fe-depleted diets (Supplementary Figure S1). These results suggest that Fe has a limited impact on weight gain in mice for the period of treatment.

### Dietary iron influences circulating N-acylethanolamine production in interaction with diet composition

**Figure 2.**
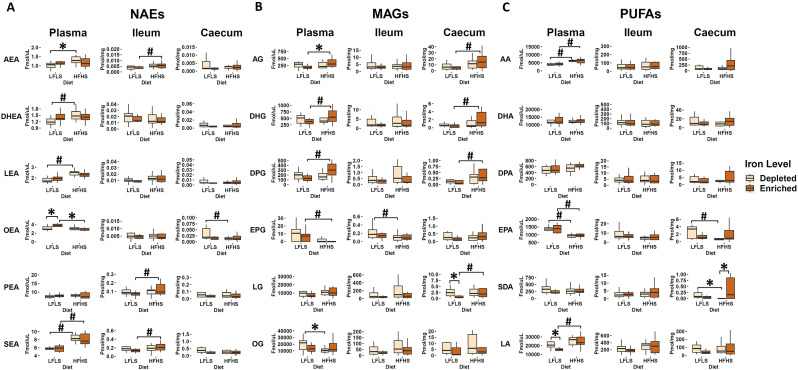
Diet and Fe modulation of endocannabinoidome mediators and some of their corresponding fatty acids. Boxplot representation of the eCBome mediators. **(**A) *N*-acylethanolamines (NAEs), **(**B) 2-monoacylglycerols (2-MAGs), and **(**C) long chain ω-6 and ω-3 polyunsaturated fatty acids (PUFAs) response to Fe-depleted and Fe-enriched LFLS or HFHS diets. Data are expressed as the mean ± SEM (n = 12). *P* values of linear contrast analysis are detailed when significant (p < 0.05) using contrast tests between enriched and depleted Fe levels, LFLS and HFHS formulations, and the combination between Fe levels and formulations. The star ‘*’ symbol was used to show the effect of Fe alone or in interaction with LFLS or HFHS. The numeral ‘#’ symbol was used to denote the effect of only LFLS or HFHS. The samples were analysed at day 28 of the study.

We quantified the eCBome mediators (NAEs, MAGs) and some of their corresponding polyunsaturated fatty acids (PUFAs) in plasma, ileum, and caecum samples (Figure 2). As observed in previous work, the eCBome response was different between plasma and the two intestinal segments studied (Guevara Agudelo et al., 2022). Macronutrient composition of the diet was the main driver of eCBome concentrations, and modulation by diet and Fe intake was not homogeneous between tissues. Overall, the influence of Fe on NAEs, MAGs, and PUFAs was always observed in interaction with the diet. Indeed, OEA showed a significant increase associated with the enrichment of Fe in LFLS diet whereas lower OEA concentrations were observed in the other conditions (Figure 2A). Circulating levels of MAGs were also modulated by Fe in interaction with diet composition. For instance, we observed that 2-AG was reduced in Fe-enriched LFLS diet compared with Fe-enriched HFHS diet. By contrast, 2-OG was increased in Fe-depleted LFLS diet compared with HFHS diet. Interestingly, significant increase of the PUFA LA was associated with the depletion of Fe in the diet in combination with LFLS (Figure 2C). Caecum SDA showed a statistically significant reduction associated with Fe depletion in HFHS diet. These results suggest a differential role of Fe intake and its interaction with dietary fat and sucrose levels in modulating the concentration of some eCBome mediators or their corresponding fatty acids. Several fatty acids and eCBome mediators were modulated solely by the diet. Circulating 2-DHG, 2-DPG, DHEA, LEA, and SEA showed an increase associated strictly with HFHS diets, while 2-EPG, as well as its precursor EPA, showed a reduction associated with HFHS diets. In the intestine, we observed that modulation of most NAEs and MAGs and their corresponding PUFAs were associated with dietary fat content and not dietary Fe levels. In the ileum, AEA and SEA were higher with HFHS than the LFLS diet. By contrast, in the caecum, EPA showed an increase with LFLS. 2-AG, 2-DHG, 2-DPG, and 2-LG levels were higher with the HFHS than the LFLS diet. Overall, these results indicate that Fe, in interaction with the macronutrient composition of the diet, influences the production of circulating NAEs, while the formulation of HFHS diets mainly increased MAGs in the intestine.

### Iron modulates the concentration of caecal prostaglandins with the LFLS diet

**Figure 3.**
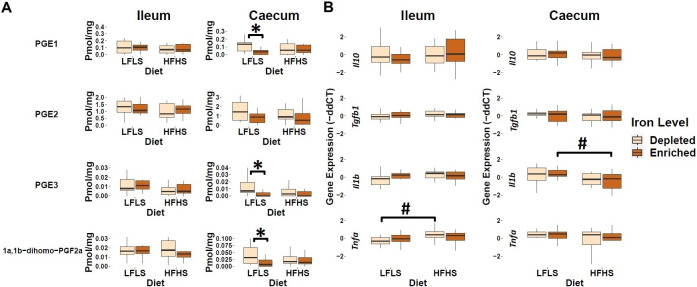
Response of intestinal prostaglandins and mRNA gene expression of immune response in Fe-depleted and Fe-enriched LFLS or HFHS diets in the intestine. Boxplot representation of the eCBome mediators in (A) ileum and (B) caecum. mRNA expression of immune response as fold change (FC) calculated using the ΔΔ*C_T_* method in Fe-depleted and Fe-enriched LFLS or HFHS diets in the intestine, ileum, and caecum. Data are expressed as the mean ± SEM (n = 12). *P* values of linear contrast analysis are detailed when significant (p < 0.05) using contrast tests between enriched and depleted Fe levels, LFLS and HFHS formulations and the combination between Fe levels and formulations. Gene expression was normalized to *Hprt.* The star ‘*’ symbol was used to show the effect of Fe alone or in interaction with LFLS or HFHS. The numeral ‘#’ symbol was used to denote the effect of only LFLS or HFHS. The samples were analysed at day 28 of the study.

In addition to endocannabinoid congeners and PUFAs, we also quantified lipid mediators that could respond differentially to dietary intake of Fe. In this sense, we evaluated the response of PGE_1_, PGE_2_, PGE_3_, and 1a,1b-dihomo PGF_2α_ (Figure 3). In the caecum, we observed that the enrichment of Fe in the diet decreased the levels of prostaglandins PGE_1_, PGE_3_, and 1a,1b-dihomo PGF_2_α. PGE_2_ exhibited a similar trend of reduction with Fe enrichment but did not display a significant difference. We did not observe this effect in the ileum (Figure 3A). In the intestine, the expression of genes involved in inflammation was not influenced by dietary Fe intake, but rather by diet formulation (Figure 3B). For instance, in the ileum, we observed an increase of *Tnfa* expression levels only in those mice fed with the HFHS diet with depleted Fe, while in the caecum this increase was not evident. In addition, the expression of *Il1b* was significantly increased by the LFLS diet in caecum. Taken together, these results point to a possible role of Fe intake in intestinal immune response by modulating the production of bioactive lipids such as prostaglandins, although with limited effect on the intestinal expression of inflammation-associated genes.

### Iron affects specific microbial species in interaction with the diet

**Figure 4.**
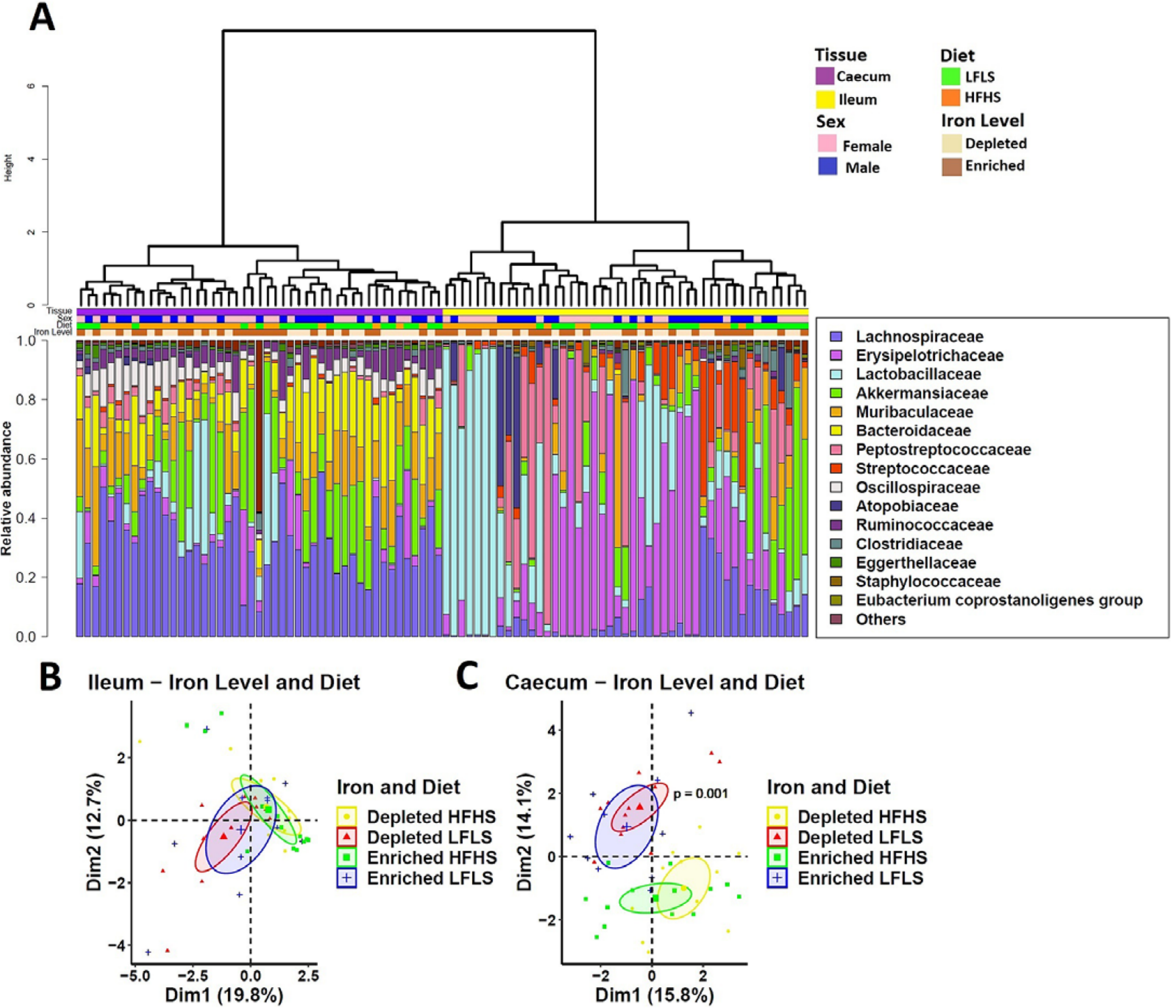
Intestinal microbiota composition in response to Fe-enriched and Fe-depleted LFLS or HFHS diets. (A) Relative bacterial abundance at the family level in response to Fe-enriched and Fe-depleted LFLS or HFHS diets in ileum and caecum. Families representing less than 1% of total bacterial abundance were aggregated. Dendrogram showing hierarchical clustering based on Canberra distance between samples determines the sample order. The corresponding annotations for tissue, sex, diet, and Fe level are displayed. Principal component analysis shows the impact of Fe-depleted/enriched and LFLS/HFHS diets on gut microbiota composition in the **(**B) ileum, and **(**C) caecum. PERMANOVA indicates the significance of microbiota composition between the dietary conditions. The samples were analysed at day 28 of the study.

**Figure 5.**
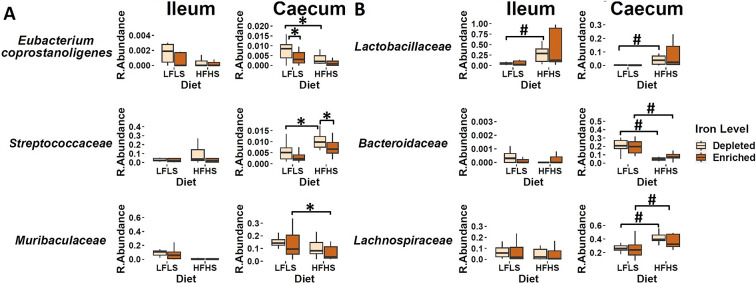
Effect of Fe-depleted and Fe-enriched LFLS or HFHS diets on bacterial relative abundance at the family level in the ileum and caecum. (A) Effect of Fe in the interaction of LFLS and HFHS formulations on intestinal microbial families. (B) Effect of solely LFLS or HFHS formulations. Data are expressed as the mean ± SEM (n = 48). *P* values of linear contrast analysis are detailed in the bottom when significant (p < 0.05) using contrast tests between enriched and depleted Fe levels, LFLS and HFHS formulations, and the combination between Fe levels and formulations. The star ‘*’ symbol was used to show the effect of Fe alone or interaction with LFLS or HFHS. The numeral ‘#’ symbol was used to denote the effect of only LFLS or HFHS. The samples analysed and showed are at day 28 of the study.

We investigated whether specific gut microbial families responded differentially to dietary Fe enrichment and whether these associations were dependent on fat and sucrose intake. As observed previously (Guevara Agudelo et al., 2022), the intestinal microbiota composition showed a remarkable differentiation between the segments of the intestine (p < 0.01, PERMANOVA) (Figure 4A). Interindividual differences in microbial taxa were more pronounced in the ileum than in the caecum, which was more homogeneous. Thus, in the ileum, microbiome did not show a clear influence of Fe or diet (Figure 4B), while in the caecum the difference was evident between LFLS and HFHS diets (Figure 4C). Three intestinal microbial families (*Eubacterium coprostanoligenes group*, *Streptococcaceae*, and *Muribaculaceae*) responded directly to Fe intake or interaction between Fe and diet content exclusively in caecum, as in the ileum no microbial family responded to the dietary changes in Fe, be it alone or in interaction with diet. For instance, *E. coprostanoligenes group* bacteria showed an increase in its relative abundance associated with the interaction of Fe-depletion with LFLS diets. Similarly, the relative abundance of *Streptococcaceae* was higher with the interaction between the depletion of dietary Fe with the HFHS formulation (Figure 5A). Concomitantly, *Muribaculaceae* showed a slight increase in its relative abundance due to the interaction of Fe-enrichment with LFLS formulations. Other microbial families in the ileum such as *Lactobacillaceae* responded to the macronutrient content of the diet and exhibited a higher abundance with HFHS diets. *Bacteroidaceae* was more abundant with the LFLS diet, and *Lachnospiraceae* was increased with HFHS diet (Figure 5B). Interestingly, increased abundance of both microbial taxa occurred only in Fe-depleted diets. Taken together, these results indicate that Fe, in interaction with diet formulation, shifted specific microbial families in an intestinal segment-dependent manner.

### Dietary Fe in interaction with sex influences circulating N-acylethanolamines, cytokine gene expression, and specific intestinal bacteria

**Figure 6.**
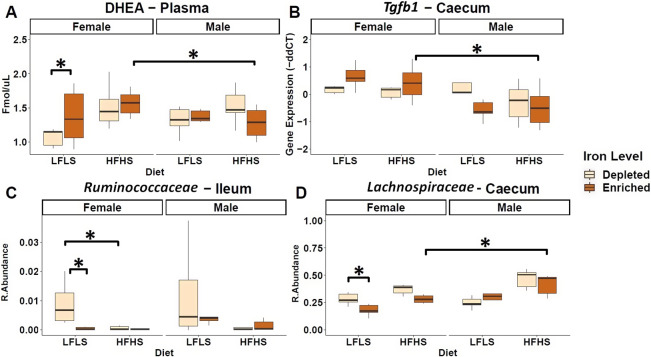
Iron influences in a sex-dependent manner circulating *N*-acylethanolamines (NAEs), cytokine gene expression, and intestinal microbial species. (A) Boxplot representation of the NAEs in plasma, (B) mRNA expression of *Tgfb1* as fold change (FC) calculated using the ΔΔ*C_T_* method, (C) relative abundances of *Ruminococcaceae* in the ileum and (D) *Lachnospiraceae* in the caecum. Data are expressed as the mean ± SEM (n = 48). *P* values of linear contrast analysis are marked with a star ‘*’ when significant (p < 0.05) using contrast test between enriched and depleted Fe levels, LFLS and HFHS formulations, the combination between Fe levels and formulations, and the sex of the animal.

In addition to the impact of dietary Fe and diet formulations, we studied the effect of Fe and its interaction with the sex of the animals regarding the changes in the production of eCBome mediators, intestinal cytokine gene expression and gut microbiota species. We found that circulatory levels of DHEA showed an interaction between Fe-depletion and the sex of the animal, such as reduced DHEA levels in Fe-depletion were observed only in females (Figure 6A). Concomitantly, we found a significant increase in the expression levels of *Tgfb1* in females compared with males under the interaction between the Fe-enriched diets with the HFHS formulation (Figure 6B). Similarly, microbial families such as *Ruminococcaceae* and *Lachnospiraceae* exhibited a reduction in their relative abundance associated with the interaction between Fe enrichment in diets and female mice. In addition, the reduction of Fe in the diets increased the relative abundance of specific microbial families in female mice such as *Lachnospiraceae* and *Ruminococcaceae.* Furthermore, *Lachnospiraceae* family showed an increase in males over females with Fe-enriched diets and the HFHS diet.

## Discussion

In this study, we investigated the effect of Fe depletion (12 mg/kg) and enrichment (150 mg/kg), in interaction with macronutrients (LFLS or HFHS), on the eCBome and gut microbiota in a mouse model susceptible to obesity. In contrast with several studies that used bleedings or diets with less than 6 mg Fe/kg to characterize the metabolic defects associated with severe Fe deficiency (B R Blakley, 1988; Cooksey et al., 2010; Santos et al., 1998), we did not target severe dietary Fe depletion, and the model used here did not provoke either anaemia in the Fe-depleted diets or hemochromatosis in the Fe-enriched diets. The present study could be considered short-term as it was only 4-weeks long. Although our aim was not to produce systemic and tissue inflammation, previous studies have shown that 4 weeks of an obesogenic diet in mice is enough to alter the inflammatory phenotype and provoke changes in gut microbiota (Cani et al., 2009; Guevara Agudelo et al., 2022).

The results obtained here suggest that short-term Fe administration may have little direct effect on body weight modulation, since there was no weight gain associated with either the enrichment or depletion of Fe during the length of the study. The fact that caloric intake did not increase significantly after Fe supplementation could explain the lack of Fe-associated weight gain. It is possible that the interaction between Fe and other nutrients may have affected weight gain in comparison with other studies using different diets (Lynch and Cook, 1980; Piskin et al., 2022). In this regard, Fe absorption may be influenced by the presence of other nutrients in the diet, which were not investigated. Yet, there is increasing evidence that obesity and Fe status are connected (Cepeda-Lopez et al., 2013; Kitamura et al., 2021), as human studies report a reduction in Fe plasma levels with increasing adiposity (Manios et al., 2013; Seltzer and Mayer, 1963; Wenzel et al., 1962). Indeed, long-term treatment of Fe deficiency anaemia for 4–6 months by increasing Fe gradually induced weight loss (Aktas et al., 2014). Besides, it has also been reported that Fe supplementation for 15 weeks reduces diet-induced weight gain (Kitamura et al., 2021), with significant changes being observed from 12 weeks of treatment.

Accumulating evidence has revealed a strong link between dietary Fe and lipid metabolism (Cunnane and McAdoo, 1987; Zhou et al., 2011). In fact, fatty acid composition in tissues can be modified as a consequence of nutritional Fe deficiency (Johnson et al., 1989). Furthermore, the role of Fe in fatty acid desaturation has been demonstrated (Romero et al., 2018). Previous studies have also shown the production of ω-6 PUFAs, particularly LA, can be regulated by dietary Fe levels (Ananda Rao et al., 1980). Concordantly, we found a significant increase in LA associated with dietary Fe-enrichment in combination with LFLS (Figure 2C). We evaluated the effect of Fe intake in interactions with low or high calorie diets on the circulating and intestinal levels of the most studied eCBome mediators (NAEs and MAGs), and some of their corresponding PUFAs. The eCBome is known to be highly influenced by dietary intake as well as by body composition (Castonguay-paradis et al., 2020). It is understood to play an important role in physiological processes related to metabolic health (Di Marzo, 2018). In this study, the influence of Fe on NAEs, MAGs, and PUFAs was always observed in interaction with diet formulations. Indeed, OEA showed a significant increase associated with the enrichment of Fe in LFLS diet only, with the other conditions having lower and comparable OEA concentrations (Figure 2A). As expected, circulating levels of AEA were higher with the HFHS diet (Lacroix et al., 2019), but interestingly this difference was more pronounced in interaction with Fe-depletion, as it was for DHEA. Recently, there has been growing interest in a group of NAEs that are congeners of AEA but that seem instead to act through mechanisms independent of cannabinoid receptors. This group includes the monounsaturated analogue OEA (Piomelli, 2013; Romano et al., 2014), which share biosynthetic and catabolic pathways with AEA (Okamoto et al., 2004) but exerts contrary effects on the regulation of food intake and lipid metabolism. Unlike AEA, OEA has no binding affinity to the CB_1_ receptor (V M Showalter et al., 1996) and its administration reduces food consumption in rodents. Supplementation with Fe has been associated with increased appetite and food intake independently of weight gain (Gao et al., 2015). In this study, we found a significant increase in OEA levels associated with Fe enrichment in the LFLS diet, although there was no significant correlation between circulating OEA levels and food intake (p = 0.81, Spearman correlation). Recent studies have shown that OEA acts as a gut-derived satiety factor (De Filippo et al., 2023; Gaetani et al., 2010) and might be involved in eating disorders (Gaetani et al., 2008), obesity (Matias et al., 2012) and type 2 diabetes (Annuzzi et al., 2010). Among other functions, OEA controls the secretion of GLP-1, suggesting a synergistic action of this NAE with intestinal microorganisms in the regulation of several homeostatic functions, since GLP-1 has numerous metabolic actions including decreased gastric clearance, inhibition of food intake, and stimulation of glucose-dependent insulin secretion (Müller et al., 2019). Results of this study suggest that Fe intake may modulate circulating OEA levels and this points out to the possibility of dietary interventions to increase levels of this mediator and, hence, affect its main receptors, that is the peroxisome proliferator-activate receptor α (PPARα), the transient receptor potential vanilloid of type 1 (TRPV1) channel, and the G-protein-coupled receptor 119 (GPR119), all of which are known to counteract obesity (Christie et al., 2018; Grimaldi, 2001).

Several 2-MAGs, including 2-AG, 2-DPG, and 2-DHG, showed higher concentrations with Fe-enriched HFHS diet compared with Fe-enriched LFLS diet both in plasma and in the caecum, but not in the ileum. These mediators have been linked to the modulation of metabolic activity and inflammation (Barrie and Manolios, 2017; Hillard, 2017; Poursharifi et al., 2017). Intestinal 2-MAG metabolism is tightly linked to re-esterification to triacylglycerol and crosstalk between Fe and lipid pathways, including alterations in cholesterol, sphingolipid, and lipid droplet metabolism in response to Fe levels have been reported (Chon et al., 2007; Rockfield et al., 2018). In a previous study, we investigated the impact of the trace mineral selenium (Se) on the eCBome (Guevara Agudelo et al., 2022). Although Se had a significant effect on weight gain particularly under LFLS diet, it showed an opposite effect to Fe in its impact on intestinal 2-MAGs levels. Notably, the levels of mediators such as 2-AG, 2-DHG, and 2-DPG in the caecum were favoured in Se-depleted HFHS diets, whereas, in the present study, we observed that these mediators were increased by the HFHS diet only in the presence of Fe supplementation (Figure 2B). Given the association between tissular 2-AG levels and dysmetabolism, observed also in humans (Silvestri and Di Marzo, 2013), it is tempting to speculate that individuals may be protected by the negative effects of a cafeteria-type diet with supplementation of Se and slight reduction of dietary Fe.

Fe is an important cofactor involved in the synthesis of AA, which plays functions associated with cell signalling and serves as a precursor of numerous oxygenated derivatives such as prostaglandins. The fact that we have identified increased circulating PUFAs and 2-MAGs is consistent with a Fe proposed involvement in immune response (Nairz and Weiss, 2020). Indeed, during Fe-supplementation, increased release of AA and eicosanoids have been associated with lipid oxidation reactions (Peterson et al., 1978), and prostaglandin metabolism (Mattera et al., 2001; Wright and Fischer, 1997). We observed reduced levels of PGE_1_ and PGE_3_ as well as a trend for reduced levels of PGE_2_ in the caecum with the Fe-enriched LFLS diet, suggesting lowered inflammation in this tissue. These effects were not observed in the ileum, which possibly reflects the lack of changes observed in this tissue of the biosynthetic precursors of PGE_2_ (AA and possibly 2-AG) and of PGE_3_ (EPA and possibly 2-EPG) and the increase of the pro-inflammatory cytokine, *Tnfa*, with the Fe-depleted HFHS.

Bioavailability of Fe in the gut lumen also plays an important role for the microbes that reside in this dynamic environment (Seyoum et al., 2021). Competition for its acquisition takes place at the intestinal host-bacteria interface (Nairz et al., 2010; Yilmaz and Li, 2018). Fe availability is known to be critical for bacterial growth, and Fe starvation is an effective strategy to limit bacterial survival. Nutrients from the diet are absorbed in different sections of the intestine (Kiela and Ghishan, 2016), which promotes specific microbial niches (Pereira and Berry, 2017). In microorganisms, Fe serves as a cofactor for proteins involved in key microbial metabolic pathways such as redox reactions, DNA synthesis, and the production of short-chain fatty acids (SCFA) (Dostal et al., 2015) and, subsequently, the proliferation and growth of almost all microbiota, including both the commensal and pathogenic species, are dependent on the utilization of unabsorbed dietary Fe. We report, here that a limited number of microbial families exhibited different relative abundances based on Fe and macronutrient intake. While intestinal microbiota composition displayed a remarkable differentiation between the segments of the intestine, differences in microbiome composition associated with Fe intake were observed in the caecum but not in the ileum. Here we found that the *Eubacterium coprostanoligenes group*, a cholesterol-reducing intestinal bacterium that synthesizes coprostanol (Juste & Gérard, 2021), and *Streptococcaceae* both show an increase in their relative abundance following Fe-depletion but under different macronutrient combinations. This suggests the presence of macronutrients may be necessary for the adaptation of some microbial species to the changes in the bioavailability of intestinal micronutrients (Sung et al., 2023). By contrast, we found that *Muribaculaceae* shows an increase in its relative abundance during Fe-enrichment in combination with LFLS diets, which had already been observed in a previous study (Ippolito et al., 2022). Other microbial families, including *Lactobacillaceae*, *Bacteroidaceae*, and *Lachnospiraceae*, responded exclusively to dietary formulations and not to Fe intake (Figure 5B). Interestingly, *Lactobacillus* was found by Dostal and collaborators to be modulated by Fe in mice given a chow diet (Dostal et al., 2012), strengthening the idea that the interaction between micronutrients and macronutrients is a key element in microbiome modulation. Taken together, these results highlight the fundamental shaping factor exerted by diet on intestinal populations. Finally, although dietary components other than Fe levels were determinants for the differential production of eCBome mediators and intestinal microbial families, the sex of the mice also impacted both systems, which is consistent with previous findings (Guevara Agudelo et al., 2022).

## Conclusions

Overall, our results indicate that the macronutrient composition of the diet modulates the response of the eCBome and the microbiome to Fe intake in mice, a phenomenon that was also observed for selenium, another trace mineral (Guevara Agudelo et al., 2022). Specifically, an increase in circulating levels of OEA was associated with Fe enrichment in the LFLS diet, concomitantly with decreased concentrations of plasma LA, caecal prostaglandins, and the caecal abundance of *E. coprostanoligenes*, potentially in reaction to Fe availability in a less dietary rich environment. By contrast, the Fe-depleted HFHS diet showed an elevation of AEA, which is usually associated with negative metabolic health outcomes. This suggests a crosstalk between the amounts of trace minerals and the dietary macronutrient content to generate a differential impact on the levels of eCBome mediators and their potential role in metabolic complications. In conclusion, our findings show that Fe might, in interaction with the diet, modulate intestinal processes as well as the host response to dietary stress. This study demonstrates how complex is the interplay between dietary components, the gut microbiota ecosystem, and host lipid signalling systems. The present findings should open the path for mechanistic studies exploring the molecular basis of the impact of macronutrients on the gut microbiome–eCBome axis, in response to Fe deficit or supplementation, and the role of this interaction in low-grade inflammation such as that accompanying diet-induced obesity.

## Supporting information

Guevara Agudelo et al. supplementary material Guevara Agudelo et al. supplementary material

## Supplementary material

The supplementary material for this article can be found at http://doi.org/10.1017/gmb.2025.1.
