# Supplementary material for: Dietary iron interacts with diet composition to modulate the endocannabinoidome and the gut microbiome in mice
Source: Gut Microbiome (Camb). 2025 Feb 14;6:e12. doi: 10.1017/gmb.2025.1 (PMC12277099; doi:10.1017/gmb.2025.1)
Supplement: Guevara Agudelo et al. supplementary material [file S2632289725000015sup001.docx]

**
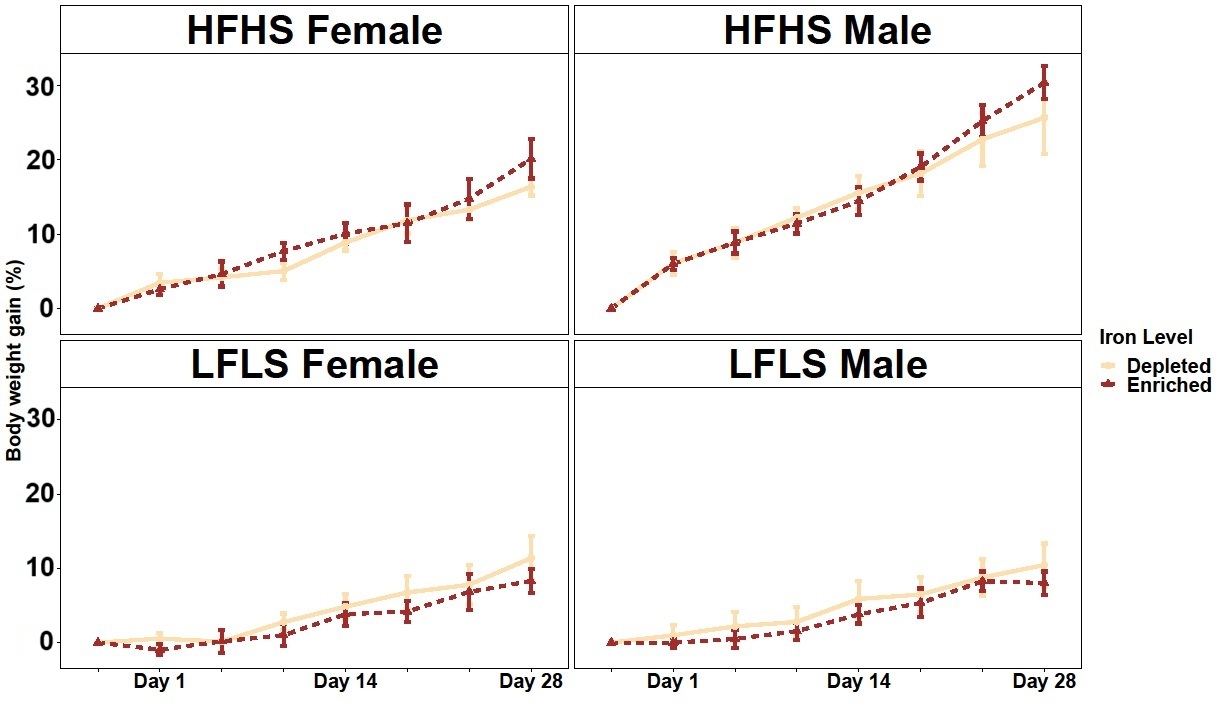
**

**Figure S1: Weight gain in female and male mice fed with fed Fe-enriched and Fe-depleted LFLS or HFHS diets**. Groups of 12 mice (6F/6M) were fed Fe-enriched and/or Fe-depleted diets for 28 days. Generalized linear regression models were used to identify the effects of time or Fe and interactions. Data are expressed as mean ± SEM (n = 6).

**
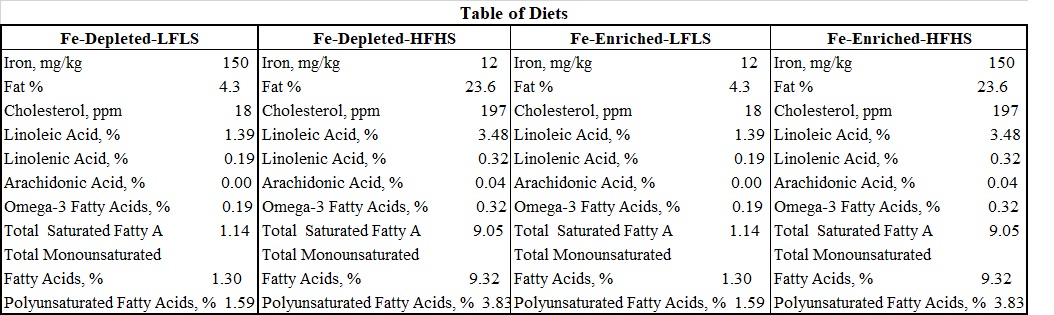
**

**Table S1: Lipid and Fe content of diets.**
